# Development and Initial Evaluation of the Web-Based Self-Management Program “Partner in Balance” for Family Caregivers of People With Early Stage Dementia: An Exploratory Mixed-Methods Study

**DOI:** 10.2196/resprot.5142

**Published:** 2016-03-01

**Authors:** Lizzy MM Boots, Marjolein E de Vugt, Hanneke EJ Withagen, Gertrudis IJM Kempen, Frans RJ Verhey

**Affiliations:** ^1^ School for Mental Health and Neurosciences and Alzheimer Centre Limburg Department of Psychiatry and Neuropsychology Maastricht University Maastricht Netherlands; ^2^ CAPHRI School for Public Health and Primary Care Department of Health Services Research Maastricht University Maastricht Netherlands

**Keywords:** carers, dementia, focus groups, Internet, psychosocial support systems

## Abstract

**Background:**

People with dementia increasingly depend on informal caregivers. Internet-based self-management interventions hold considerable promise for meeting the educational and support needs of early stage dementia caregivers (EDCs) at a reduced cost.

**Objective:**

This study aimed to (1) develop an online self-management program for EDC to increase self-efficacy and goal attainment, and (2) evaluate the program’s feasibility and report preliminary data on effectiveness.

**Methods:**

Based on the Medical Research Council (MRC) framework for the development and evaluation of complex interventions, a stepwise approach was adopted to explore potential user needs and develop and validate the content by means of (1) focus group discussions with dementia caregivers (N=28), (2) interviews with dementia care professionals (N=11), and (3) individual think-aloud usability tests with EDC (N=2) and experts (N=2). A pilot evaluation was conducted with EDC (N=17) to test the feasibility and establish preliminary effects. Self-report measures of feasibility were completed after the completion of intervention. Self-efficacy and goal attainment were evaluated before and after the intervention.

**Results:**

The different steps provided useful information about the needs of potential users regarding the content and delivery of the program. This resulted in the newly developed “Partner in Balance” program. At the start, system failures resulted in a high noncompleter rate (7/17, 41%), but at the end, an acceptable feasibility score of 209 (range 54-234) was found. The convenience of completing the program at home, the tailored content, and the guidance (face-to-face and online) were appraised positively. Preliminary effects on caregiver self-efficacy (*P*<.05) and goal attainment (*T*>50) were promising.

**Conclusions:**

Adaptations were made to the program to limit the amount of system failures and prevent high noncompleter rates. As recommended by the MRC framework, confirming the feasibility and preliminary effectiveness is a valuable step toward examining the effectiveness of this newly developed intervention.

**Trial Registration:**

Dutch Trial Register (NTR): NTR4217; http://www.trialregister.nl/trialreg/admin/rctview.asp?TC=4217 (Archived by WebCite at http://www.webcitation.org/6f6B8lvRP).

## Introduction

Chronic illness and decreased well-being are expected to become global public health challenges [1], with dementia being one of the most common disorders in elderly individuals [2]. With less formal health care available and more people in need of care, the caring role has now shifted to the informal caregivers at home [3]. However, caregivers of people with dementia are at an increased risk of burden, stress, and have a fourfold risk of becoming depressed compared with noncaregivers [4,5]. As such, this transition of friend/family member into the caring role increases the need for effective caregiver interventions to improve their mood and quality of life.

Although recent face-to-face caregiver interventions appeared to be promising [6,7], the increasing gap between care supply and demand calls for alternative and cheaper methods for providing education and support to informal caregivers [3,8]. Internet interventions may help caregivers cope with the challenges of caring for a person with dementia [9]. A recent literature review [10] showed that the currently available Internet interventions for caregivers of people with dementia have promising effects on their confidence and burden, given that they included multiple components and were tailored to the individual participant (caregiver). In addition, Internet-delivered caregiver support may prevent accessibility problems for informal caregivers who are isolated or have difficulties accessing traditional health care services [11,12].

At present, remote support for dementia caregivers is increasing, and new Internet interventions are being developed [13-19]. These programs are, however, mainly focused on dealing with dementia-related problems (eg, neuropsychiatric symptoms) that occur at an advanced stage of the caregiver career; by contrast, early stage interventions can prepare caregivers for their future tasks at a stage where stress and burden are relatively low [20]. Early intervention and support for caregivers have proven to be effective in reducing strain, increasing caregiver confidence, and delaying institutionalization of the person with dementia [21-23]. Moreover, early therapeutic interventions may help caregivers identify their needs based on their individual situation and facilitate the adaptation process [24]. The Stress and Coping paradigm by Lazarus and Folkman [25] and the Social Learning theory by Bandura [26] propose that taking charge of the changes in one’s life increases self-efficacy, and can therefore reduce caregiver stress and its impact on general well-being [27]. Following these theories, an early stage support program for caregivers should focus at positively managing life with dementia rather than managing the dementia itself [28]. Self-management programs suit the caring role transition and have previously been used to support informal caregivers of several chronic diseases with promising results [12,29,30].

### Iterative Development Process

**Figure 1:**
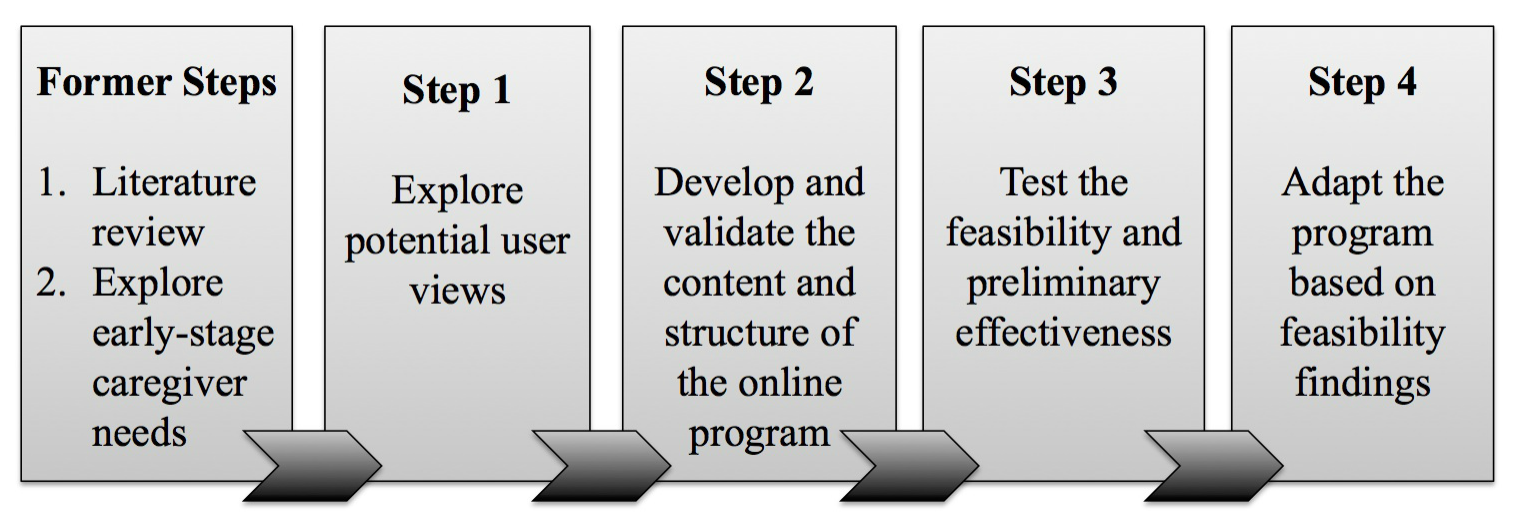
Iterative development process informed by MRC framework.

This study describes the development of an online self-management program for early stage dementia caregivers (EDC) to improve self-efficacy and goal attainment. We closely followed the iterative process of the new Medical Research Council (MRC) Framework for the development of complex interventions [31]. The first 2 steps in the intervention development are described elsewhere [10,24]. The current paper describes the next 4 steps (Figure 1) spread over a 2-year period (2012-2014). These steps are described in the following sections.

## Methods and Results

### Step 1: Explore Potential User Views: Focus Groups

#### Methods

In-depth exploratory focus group interviews were conducted to explore EDC’s views on the content and format of an early stage intervention (see Ref [24] for detailed methodology). A context-mapping approach [32] was used: a booklet examining personal Internet and computer use and a collage displaying chosen themes based on significance during the early stages. Available themes were preselected based on existing interventions, early stage dementia care literature [21,33], and expert knowledge. Blank cards were also provided. The most often selected and highest appraised themes were compiled. Focus group interviews were transcribed verbatim and analyzed independently based on deductive content analysis by 2 of the study authors (LMMB and MEdV). Topics that were mentioned frequently and explicitly served as the basis for categorization. Categories were merged into common themes in a consensus meeting (LMMB and MEdV).

#### Results

Participant characteristics (N=28) are presented in Table 1. The booklet on computer use was completed by 18 participants. Reasons for noncompletion were (1) overlooking the booklet (N=7) and (2) not understanding its value (N=3). Participants used the computer for multiple purposes, for example, finding information (15/18, 83%), email (14/18, 78%), financial transactions (14/18, 78%), writing (14/18, 78%), viewing photos (9/18, 50%), playing games (8/18, 44%), (video) chatting with family members (3/18, 17%), and shopping (2/18, 11%). However, 3 participants did not use a computer and were not inclined to do so in the near future.

During the focus groups, the majority of the participants considered Internet interventions as efficient due to the high level of accessibility, especially when feeling pressed for time or being bound to one’s home. Receiving answers to urgent queries was also considered very positive.

The advantages are no travelling time and the possibility to search what I want to know when it is convenient for me.

P9

I would like to be able to extract the information that is important for me at that particular moment, because we’re all so different. When you’re in need of an answer, a personal response would be great.

P13

Blended care (face-to-face care combined with online modules) was preferred over online care only, due to the personal contact with a professional.

People experience emotions, while a computer is just an object. Seeing the person you are talking to is really important. Once you know each other, email or telephone is fine for information exchange.

P5

**Table 1:** Background characteristics of the caregivers (N=28) and the care recipients (N=25).

Characteristics		n (%) or mean
Age, years		63.6
**Gender**		
	Men	7 (25)
	Women	21 (75)
**Relationship to the care recipient**		
	Spouse	22 (79)
	Child	2 (7)
	Child-in-law	2 (7)
	Sibling	1 (4)
	Friend	1 (4)
**Living together with care recipient**		
	Yes	21 (75)
	No	7 (25)
**Care recipient diagnosis**		
	Mild cognitive impairment	8 (32)
	Alzheimer’s disease (AD)	11 (44)
	Vascular dementia	3 (12)
	Parkinson	1 (4)
	Dementia not otherwise specified	2 (8)
**Care recipient years of diagnosis**		
	0.5	5 (20)
	1-3	9 (36)
	4-6	10 (40)
	7-10	1 (4)

All participants (N=28) completed the collage of themes during the interviews. Participants stressed the importance of an intervention tailored to the stage of the disease and the individual caregiver’s situation, with less focus on coping with dementia and negative stigmatizing information about the future. Learning how to stay healthy by positively managing one’s life and learning to accept the changes were considered important, and so is the significance of information provided by other caregivers. A more flexible choice of themes, based on personal needs and areas of interest, was considered desirable. The themes most often mentioned and highest appraised are listed in Table 2.

**Table 2:** Themes most often selected and highest appraised by family caregivers (N=28).

Themes	Selected n (%)	Appraised most important n (%)
Practical tips	27 (96)	14 (50)
Role and relationship changes	22 (79)	5 (18)
Information about the disease	21 (75)	12 (43)
Balance in activities	19 (68)	2 (7)
Focus on the positive	19 (68)	5 (18)
Communication	18 (64)	5 (18)
Acceptance	16 (57)	9 (32)
Insecurities and worrying	12 (43)	3 (11)
Social relationships and support	12 (43)	1 (4)
Emotions and tension	10 (36)	1 (4)

### Step 2: Develop and Validate Program Content and Structure

#### Consulting Dementia Care Experts

##### Methods

Individual in-depth interviews were conducted to explore dementia care experts’ views on EDC Internet support. Experts from different institutions and regions within the Netherlands were recruited via email. Inclusion criteria were (1) professional caregiver in the Dutch dementia care field, (2) daily interaction with people with dementia (PwD) and their caregivers, and (3) ample experience in supporting EDC. The number of participants (N=11) was determined by data saturation. The redundancy of themes emerged from interviews [34]. Professional backgrounds of the experts were psychiatrists (N=1), clinical neuropsychologists (N=3), registered health psychologists (N=4), occupational therapists (N=1), social psychiatric nurses (N=1), and nurse practitioners (N=1), with an average of 13.64 (SD 7.43) years of professional experience. A semistructured interview guide was developed by authors LMMB and MEdV, which was validated by author FRJV. Topics included EDC needs, relevance and feasibility of EDC support, and themes for an EDC intervention. Brief summaries of the key points were made throughout the interview to obtain participant verification [35]. All interviews were audiotaped. The content of the verbatim transcriptions of the interviews was analyzed by summarizing common themes based on deductive content analysis.

##### Results

The experts emphasized that caregiver support needs to be tailored to the dementia stage. Concerns were raised about providing early support in the absence of later-stage problems, when caregivers are not in need of help yet and could possibly reject early stage support. Experts considered education on the disease and its course as most important. Other important themes involved accepting the disease, coping with relationship changes, stress, role management, and rumination. The importance of interaction between PwD, caregivers, and environment was also stressed. Correcting or accepting care recipients’ mistakes and notifying social network can be primary stressors in daily interaction. Too much negative information not fitting the early stages, for example, behavioral problems, care homes, and end-of-life decisions, could lead to adverse reactions and should be avoided in EDC support.

Combining the themes chosen by the experts and the caregivers resulted in 9 separate themes (Table 3). The theme “practical tips” was incorporated in the module structure to provide tips thematically.

**Table 3:** Modules of “Partner in Balance” and their key points.

Module	Key points
Acceptance	Identify changeable and unchangeable situations
	Adapt expectations and learn to let go
Balance in activities	Change in daily and pleasant activities
	Identify personal carrying capacity and burden
Communication with family member and environment	Communication changes due to memory problems
	Effective communication with adaptations
Coping with stress	Relationship between stress and health problems
	Identify and cope with stress in daily life
Focusing on the positive	Identify activities and situations that are still possible
	Find alternatives and accept adaptations
Insecurities and rumination	Recognize rumination signals and control thoughts
	Prevent looking ahead; live in the moment
Self-understanding	Self-evaluation in caregiver encounters
	Personal strengths and areas of improvement
The changing family member	The changing memory and behavior
	Influence of memory decline on daily life together
Social relations and support	Value and maintenance of social relations
	Types of support

#### Content Proposal: Existing Evidence and Conceptual Frameworks on Self-Care

##### Methods

Intervention content was proposed by authors LMMB and MEdV based on a literature review [10], EDC needs [24], identified themes in Step 1, and conceptual frameworks on self-management. The Stress and Coping paradigm [25] served as the theoretical basis for the content of the modules. According to this model, stress is experienced when a person perceives that the demands (caring for a person with dementia) exceed their personal and social resources. Caregivers’ responses to their stress situation might be mediated by their understanding of the situation and their beliefs about their ability to cope. The latter fits Bandura’s [26] concept of self-efficacy (belief in one’s capabilities). Consistent with this theory, models of dementia management emphasize the need to maintain self-worth and control [28]. An intervention aimed at increasing self-efficacy should not only educate the caregiver, but should also foster self-management by combining education with problem-solving skills, and work toward a change in behavior [36].

##### Results

The proposed self-management intervention program “Partner in Balance” (PiB) encourages caregivers to actively manage their lives and identify solutions for their specific needs [37]. Increasing knowledge, identifying and setting goals, and learning skills to achieve these previously set goals served as the basis for the intervention program. Module content was focused on role management (eg, balancing activities in daily life) and emotional management (eg, dealing with fear and insecurity about the future) [38]. Formulating, planning, and executing personal goals can be learned using a proactive 5-step change plan (Textbox 1) often used in self-management [38], which was integrated into each module. By formulating and planning a personal change plan, caregivers learn to anticipate on stressful situations and gain confidence in their ability to take care of the situation and themselves [38]. Because caregivers greatly varied in their needs, personal goals, and interest, a flexible choice of modules was used. Successful elements that were identified in the literature review [10], including tailored caregiving strategies and contact with a coach and/or other caregivers, were included in the program content likewise.

**The five self-management steps applied in each module.**

Step 1: Recognize areas that you wish to change or to maintain

Step 2: Recognize additional conditions and barriers

Step 3: Generate alternative strategies for the problem(s)

Step 4: Write down your final plan SMART (*s*pecific, *m*easurable, *a*ttainable, *r*ealistic, and *t*imely)

Step 5: Evaluate when you will be satisfied with your progress

#### Validating Proposed Program Content

##### Methods

Dementia care experts (N=4) not involved in the initial interviews were asked to read the material and provide comments with respect to language, tone, amount of information, and significance and propriety of the content. They worked as clinical neuropsychologist (N=1), registered health psychologist (N=1), occupational therapist (N=1), and social psychiatric nurse (N=1), with an average of 9.75 (SD 2.22) years of professional experience.

##### Results

The experts provided comments concerning the content of each module. Stigmatizing, complex, or unclear language was reported and alternatives were provided. The textual content of the program was adapted accordingly.

### Step 3: Testing the Feasibility and Preliminary Effectiveness

#### Think-Aloud Usability Testing

##### Methods

A Web-based fully operational program was developed based on the senior-friendly website checklist [39]. Initial usability flaws were tested with the “think-aloud” method [40]. Potential users were asked to think aloud while using the system, allowing the researchers to understand the reasons behind their usage behavior. Participants (N=4) were randomly selected from the focus group interviews (N=2) and the expert interviews (N=2). All aspects of the senior-friendly website guidelines were checked (eg, font size, contrast, menus and navigation, button style and size, phrasing, illustrations and videos, and Web assistance) and additional comments on user-friendliness were explored in-depth. The interviews were audiotaped and the verbatim transcripts were combined with field notes made by author LMMB on experienced difficulties during the walk-through. A coding scheme based on the interview protocol was used [40].

##### Results

All participants (N=4) commented on the layout, font size, contrast, tone, and navigation. The website layout was considered professional and attractive, although a uniform composition of all pages was proposed to foster cohesion. Alterable font sizes and increased contrast with the background color were suggested. Vignettes of caregivers were considered useful, but addressing people with their given name instead of their family name was considered more appealing. The tips from other caregivers were perceived as crucial. It was suggested to conclude every module with these tips to increase layout uniformity.

#### Piloting Feasibility

##### Methods

An uncontrolled pre-post-intervention pilot study with EDC was conducted to establish feasibility, as this is recommended before moving on to a larger scale effect study [31,41]. The Medical Ethics Committee of the Maastricht University Medical Centre approved this study (No NL44475.068.13, Dutch Trial registration number NTR4217). Caregivers were included in the study if they (1) were spousal caregivers of people with mild cognitive impairment [42] or mild dementia of all subtypes [43], and (2) had access to the Internet. Exclusion criteria were (1) insufficient cognitive abilities to engage in the online self-management program, (2) overburdening, (3) severe health problems (determined by the study staff), or (4) caring for PwD caused by human immunodeficiency virus, acquired brain impairment, Down syndrome, Huntington’s chorea, or alcohol abuse. Participants were recruited at memory clinics and ambulatory mental health clinics. Based on comparable feasibility studies, we aimed to include 10 participants [44,45]. Of those contacted, 17 of 43 caregivers (40%) were willing to participate and signed the informed consent form.

Feasibility was evaluated face-to-face at the caregiver’s home by a semistructured interview developed for this study—the Program Participation Questionnaire (PPQ). The PPQ was based on measurement scales for perceived usefulness and ease of use and overall acceptance of information technology [46,47] and included 30 items on usability, clarity, comfort with, and acceptability of the format on a 7-point Likert scale ranging from 1 (strongly disagree) to 7 (strongly agree). For the individual PPQ items, see Multimedia Appendix 1. Mean scores were calculated with descriptive statistics. Because there were no external criteria to properly define feasibility [48], we followed the conventional strategy of using the median score of the questionnaire as a cutoff. This approach was previously adopted in a Delphi study as evidence of agreement of intervention feasibility [49]. Based on the PPQ scale (range 54-234, median 144), scores of 145 or higher were considered “acceptable feasibility.” Mean item scores (range 1-7) will be used to make decisions on positively and negatively appraised aspects of the program. Mean item scores of 5 (slightly agree) or higher will be considered positive, mean item score below 4 (slightly disagree or lower) will need further revisions. Participants were asked to elaborate their scores. Comments were audio recorded and transcribed verbatim. Meaningful data units based on the PPQ items were identified and derived independently from the qualitative data by authors LMMB and HEJW with deductive content analysis. Furthermore, the actual accessed data use of the program (number of log-ins and features used) was compared with self-reported data.

##### Results

The study population consisted of 17 participants, of whom 10 completed the postintervention assessment. Participants who did not complete the postintervention assessment were replaced to meet the sample size suggested by previous studies [44,45]. The main reasons for not completing the program and the postintervention assessment were difficulties with the online aspect of the program (N=4), private circumstances (N=2), and disagreements with the care recipient (N=1). Completer and noncompleter characteristics are listed in Table 4.

**Table 4:** Participant characteristics for completers and noncompleters of the intervention.

Characteristics		Completers (N=10)	Noncompleters (N=7)
Age caregiver, mean (SD)		68.10 (6.54)	67.43 (5.65)
Age care recipient (people with dementia [PwD]), mean (SD)		69.90 (4.33)	71.57 (8.46)
Hours care per week, mean (SD)		44.20 (56.85)	76.43 (71.98)
**Gender, N (%)**			
	Male	7 (70)	2 (29)
	Female	3 (30)	5 (71)
**Education, N (%)**			
	High school	1 (10)	3 (43)
	College	7 (70)	4 (57)
	Graduate school	2 (20)	0 (0)
**PwD diagnosis, N (%)**			
	Mild cognitive impairment	7 (70)	5 (72)
	Alzheimer’s disease	3 (30)	1 (14)
	Vascular dementia	0 (0)	1 (14)

^a^Noncompleter rate=41.2%.

The PPQ showed a good internal consistency (alpha=.89) and had a mean sum score of 209 (SD 22.14). Given the threshold of 145 or higher, this score indicated an acceptable feasibility. Mean item scores above 5 (slightly agree or higher) were found for convenience of completing the program at home (5.9, SD 1.8), clarity of the website (6.0, SD 1.2), module structure (6.1, SD 0.6) and content (6.6, SD 0.5), privacy (6.6, SD 1.3), tailored assignments (6.1, SD 1.3), guidance by the coach (6.6, SD 0.5), and general contentment (6.4, SD 0.9). A mean item score below 4 (slightly disagree or lower) was found for usefulness of the discussion forum (2.8, SD 2.7). Table 5 shows the positive, negative, and neutral themes derived from the additional comments. Self-report usage data were comparable to tracked usage data: 106.41 (SD 96.15) minutes spent per module, including scoping the website (4.5 minutes, SD 4.13), completing the assignments and change plan (79.14 minutes, SD 77.16), contacting the personal coach (15.31 minutes, SD 16.96), and visiting the discussion forum (7.46 minutes, SD 7.09), spread out over 2.38 (SD 1.38) weeks.

**Table 5:** Positive, negative and neutral evaluation of different features of Partner in Balance (N=10).

Feature	Positive	Negative	Neutral	Quotes^a^
Online	At home	Writing personal situations feels more confronting than verbalizing them	—	*I thought it was useful to have the ability to do it at my own convenience and only when I was in the mood for it.* [P10]
	Work in own time			
Website	Quick response during problems	Technical problems: login and communication		*It was pleasant that the information was presented in parts*. [P8]
	Fragmented information			
Personal coach	Indispensable support	Goal setting during intake difficult	Help of coach during goal setting necessary, not able to do it alone.	*It is important that you have someone you can rely on during the program. It stimulates you even more and you know you are not alone.* [P8]
	Feedback boosts self-confidence and motivation			
Video clips	Relatable examples	Not applicable to every individual	Background of caregivers unclear	*I could not identify with the video clips because it was not clear who was talking and I cannot relate to the addressed problems just yet.* [P3]
	Personal aspect			
		Confronting		
Discussion forum		Too difficult to use	Coaches could feed the forum, ask questions or outline situations	*I am not going to write something out of the blue on a discussion forum. You might say the wrong things, even if you have good intentions.* [P2]
		Purpose unclear		
		Difficult to start conversation		
		No nonverbal communication		
Change plan	Useful	Felt like an obligation	Difficult to set personal goals	*The self-management assignment teaches you a lesson. How are you going to solve these things?* [P10]
	Increases awareness			
Application in daily life	Communication tips		Already apply tips in daily life	*The coach provided me with very good tips that I applied in daily life.* [P12]
General contentment	Boosts confidence	No focus on practical decisions possibly faced in the future		*On moments that you are feeling insecure, this program gives you some kind of confirmation. It makes you doubt yourself less.* [P10]
	Recommend to other caregivers			

^a^P (number): participants number in the feasibility study.

#### Piloting Preliminary Effects

##### Methods

Preliminary understanding of the effectiveness of the program was based on the baseline and postintervention assessment 8 weeks later, completed at the participant’s own convenience in an uncontrolled pilot study. At participant’s request, paper questionnaires were used. The Caregiver Self-Efficacy Scale (CSES) was used to measure domain-specific caregiver self-efficacy [50]. The subscales include 4 items on service use and 6 items on care management, with scores ranging from 1 (not at all certain) to 10 (very certain). We found a good internal consistency for both service use (alpha=.73) and care management (alpha=.87). Paired samples *t* tests were conducted to evaluate pre-post-intervention changes. The goal attainment scaling (GAS) [51] method was used to rate treatment-related change and to compare relative success of previously set personal goals. Baseline scores were set at −2. Postintervention scores can range from −2 (much lower than expected) to +2 (much better than expected), with a score of 0 meaning goal attained. Raw scores were transformed into an individual mean GAS score (*T* score) to determine goal attainment with a potential weight assigned to the goal(s) [52]. *T* scores of 50 or more (SD 10) indicate effective goal achievement.

##### Results

Postintervention, participants (N=10) had significantly higher scores on both the CSES care management subscale (mean 41.1, standard error [SE]=2.5; *t*_9_=−2.5, *P*=.03) and service use subscale (mean 32.6, SE=1.7; *t*_9_=−3.5, *P*=.01) compared with preintervention scores (mean 36.1 and SE=3.2, and mean 23.2, SE=3.4, respectively), although effect sizes were small (*d*=0.14 and 0.41, respectively).

In this study, 8 program completers set 13 goals in total; 2 program completers were not able to set goals due to personal difficulties verbalizing the desired change. Two goals (of 2 participants with multiple goals) could not be scored after the intervention, because the goals changed during the course of the study. In total, 8 goals were attained (2 attained, 5 higher than expected, and 1 much higher than expected), and 3 goals were unattained (1 much lower than expected and 1 lower than expected). The mean *T* score at baseline (set at the −2 level) was 27.8 (SD 3.04). The mean achieved *T* score after the intervention was 53.7 (SD 12.03). Table 6 shows the number of goals for each domain, with most goals set on communication with the care recipient (N=7), followed by maintaining positive activities together (N=2), obtaining social support (N=2), and planning time alone (N=2).

**Table 6:** Number of set goals and attainment scores (N=8).

Number of goals	Scores	
	Mean (SD)	Range
Number of set goals per participant	1.6 (1.06)	1.0-4.0
GAS^a^score at baseline	27.8 (3.04)	22.6-30.0
GAS^a^score achieved	53.7 (12.03)	30.0-70.0

^a^GAS: goal attainment scaling

### Step 4: Adapting the Program—Final Intervention

Following the iterative development process, the program was adapted according to the results obtained in the feasibility study. The discussion forum was expanded with regular posts from personal coaches with practical tips, literature, and events related to EDC. The role and background of the person in the video clips was clarified. In addition, the content of often-mentioned early stage situations and problems [24] was expanded and later-stage problems were made less prominent in the video clips. Furthermore, technical issues with logging in and communicating with the personal coach were resolved with the team of Web experts.

#### Final Intervention

PiB consists of three elements, namely, (1) face-to-face intake session with a personal coach, (2) online period guided by the personal coach (psychologist or psychiatric nurse with ample experience with dementia caregivers), and (3) face-to-face evaluation session with the personal coach.

##### Intake Session

In the intake session participants are introduced to the website and the self-management concept of the program. The coach and participant set personal goals using a motivational interviewing technique frequently used to identify change objectives and enhance intrinsic motivation [53]. Based on the discussed areas for improvement, participants select 4 of the 9 modules that were previously identified by experts and caregivers. Participants are provided with personal login codes to access their selected modules and edit their personal information. After the online period, participants will discuss their personal goals and their ability to cope with future difficulties in the evaluation session.

##### Online Period

During the online period of the intervention, participants follow the chosen modules during an 8-week period. The website consists of (1) a home page with a short description of the goal of the program, personal login option, contact information of the researcher, and the institutional affiliation (Maastricht University); (2) a personal page with a link to the chosen modules and a mailbox for exchanging emails with the personal coach; and (3) an online forum to interact with other caregivers, moderated by the researcher and personal coaches.

Every module has a fixed design of the following 4 components: (1) video clip of fellow family caregivers, (2) education, (3) self-reflection assignment, and (4) the 5-step change plan, guided by the personal coach who will provide individualized online feedback after completion of each module and offers assistance when needed. For every module, 2 weeks are reserved as a starting point. However, participants are allowed to complete the modules at their own pace as informed by the self-management approach [38]. The first week of a module addresses Components 1-4. Participants can send their assignment and the 5-step plan to their coach. The second week of every module is reserved for feedback from the coach, after which participants can adjust their 5-step plan if necessary.

##### Personal Coach

The personal coach is an experienced dementia care professional (psychologist or psychiatric nurse). Coaches will receive a 1-day training in self-management techniques and online help before the start of the intervention. They will receive experienced supervision from an experienced professional in psychology and self-management during the course of the intervention period to ensure quality and alignment of the feedback of the coaches according to self-management principle. Coaches are asked to support participants in choosing modules that fit their personal situation, help participants identify feasible goals, offer techniques to achieve goals, and provide participants with general constructive feedback on their assignments. Using a personal login code, coaches will be matched with the participants assigned to them. The CONSORT EHEALTH checklist is presented as Multimedia Appendix 2.

## Discussion

In this paper, the iterative development process of the Web-based self-management program PiB for EDC was presented. Use of the MRC framework enabled us to develop an intervention based on existing research, theoretical frameworks, and user and professional input. Including potential users during the design process enabled us to gain unique insights into usage behavior and challenges to adapt the technology to the needs of the target audience. A similar design has been successfully used in previous studies [54-56].

During the exploration phase, caregivers greatly varied in their need for information. Previous self-management studies confirm that personal caregiver needs should be used as a starting point [57]. Blended care was preferred over online care only, due to the personal contact with a professional. Previous studies support the value of this format as participants highly appreciated the connection with their coach or therapist [58] and felt more motivated to complete Web-based interventions [59]. By contrast, adding face-to-face contacts increases the costs of the online program and reducing the number of face-to-face contacts might harm treatment outcomes when online components are not used [60,61]. However, blending online modules with regular face-to-face therapy can increase the adherence and effectiveness of the treatment. Scientific validation of blended care interventions is warranted for the development and adaptation of future treatments [60] and can provide important support for the use of blended care interventions rather than online therapy only. The former can be more easily implemented by health services, therapists, and clients than online therapy, as they can be integrated into existing treatment and care settings [62].

Our results showed acceptable rates of satisfaction with PiB. Caregivers greatly appreciated the use of online resources due to the convenience of completing the program from their homes, which is in line with previous studies on Web-based caregiver interventions [46,63-65]. Furthermore, PiB supported the participants in the process of caregiving and boosted their self-confidence, probably due to the combination of support, self-management, and a tailored approach [66,67]. However, the noncompleter rate was high due to initial technical difficulties, which were resolved later on in the study. Unfamiliarity with using the website also caused difficulties among the older age group, resulting in a relatively young sample. A recent study confirmed that younger dementia caregivers were more likely to use the Internet for health-related purposes [68]. However, other research showed that homebound older adults with limited computer skills who receive computer training at the start of an intervention can participate without difficulties [69]. In addition, including more potential users in the thinking-aloud procedure could be helpful [40].

The discussion forum was negatively reviewed and hardly used, due to the unclear purpose, the anonymity of participants, and the high threshold for starting a conversation. However, other studies suggest that forums can serve as a valuable addition to share experiences and support [39,45,70]. Adding new tips or developments for caregivers could increase the use of a forum because the aspect of reading posts was already considered useful [70]. Furthermore, participants reported struggles with goal setting. This could be due to the relatively lower objective burden of EDC, compared with caregivers of people in the later stages of the disease. EDC might experience more subjective burden, which is more difficult to translate into specific needs. Problems with accepting and adapting to their new role may also have hindered goal setting [24]. Therefore, goal setting for EDC should focus on the enhancement of positive overall experiences and facilitation of the personal adaptation process, rather than exclusively aiming for change. This approach may help to reduce or prevent negative consequences of caregiving (eg, overburdening) at a later stage [24].

Preliminary effects on caregiver self-efficacy and goal attainment were small, yet positive. This finding is in line with Bandura’s theory on self-efficacy, which states that caregivers’ objective understanding of the situation and belief in one’s capabilities (ie, the self-efficacy level) can increase if provided with the right tools [26]. Furthermore, this finding is congruent with previous research on online support for dementia caregivers [21,71,72].

The program in this study was evaluated in a homogenous group of primary caregivers (eg, spousal caregivers), with specific attention for the spousal relationship. However, the themes may apply to a broader target group, as demonstrated by previous studies [13-19]. PiB could potentially be suitable for other primary carers, which should be further investigated in the upcoming effect study using a larger sample.

### Limitations

The small sample size, the lack of a control condition, and a possible sampling bias based on caregivers with access to the Internet make it difficult to generalize the results. However, previous studies adopting a development and feasibility approach have used similar methodology and sample sizes [15,73], fitting the purpose of formative research [31]. In addition, users and experts were closely involved in the development of the intervention, and the content and adaptations relied on in-depth participant and expert feedback. Future research should consider inclusion of caregivers in the proposed content validation, to ensure potential user feedback in every step of the development. Furthermore, drawing conclusions from the adopted median cutoff score, which is an arbitrary value, may not be justified. However, in this study, the overall feasibility score was not leading. Mean item scores were used to make program improvements.

This study used paper questionnaires. Although seniors’ use of the Internet is expected to increase over time [74], dementia caregivers seem to be less active in health-related Internet use compared with the population at large [68]. The high noncompleter rate resulted in missing post-test data from the noncompleters as these were collected after the last module. However, reasons for noncompletion and characteristics of noncompleters were provided, giving insight into their possible motives. Future effectiveness studies should include noncompleter data after the treatment and at follow-up and consider using Web-based questionnaires, with the advantages of low costs, no missing data or data entry errors, and time flexibility [75].

### Conclusion

Our tailored intervention approach appeared to be feasible for informal EDC and provided them with important support when dealing with the difficulties of caregiving. Feasibility results were used to improve the intervention. Confirmation of the feasibility and preliminary effectiveness is a valuable step toward examining the effectiveness of this intervention, as recommended by the MRC framework [31]. The PiB course is currently (November 2015) available for caregivers who are interested in participating in the effectiveness study. At the course website (Partner in Balans) they can express their interest by emailing the researcher, after which they will receive additional information about the course and the effectiveness study.

## Appendix

Multimedia Appendix 1 Program Participation Questionnaire.

Multimedia Appendix 2 CONSORT-EHEALTH checklist V1.6.2 [76].

## Abbreviations

CSES: Caregiver Self-Efficacy Scale
EDC: early stage dementia caregivers
GAS: goal attainment scaling
MRC: Medical Research Council
PiB: Partner in Balance
PPQ: Program Participation Questionnaire
PwD: people with dementia
SE: standard error
