# Supplementary material for: Development and Initial Evaluation of the Web-Based Self-Management Program “Partner in Balance” for Family Caregivers of People With Early Stage Dementia: An Exploratory Mixed-Methods Study
Source: JMIR Res Protoc. 2016 Mar 1;5(1):e33. doi: 10.2196/resprot.5142 (PMC4795319; doi:10.2196/resprot.5142)
Supplement: Multimedia Appendix 1 [file resprot_v5i1e33_app1.pdf]

Program Participation Questionnaire

**Rate each item on a scale from 1 (strongly disagree) to 7 (strongly agree)**	**Please elaborate your item score**
1. It was convenient to follow the program online	*- What made it (not) convenient?*
2. The website was easy to use	*- What was specifically easy/difficult?*
3. I was able to read the texts perfectly	*- Comments on font, size, contrast?*
4. The website indications were clear to me	*- Why were they (not) clear?*<br>*- Points for improvement?*
5. The amount of information presented was good	*- Video clip length, amount of text*
6. The content of the modules was clear	*- Comprehensible?*<br>*- Points for improvements?*
7. I was satisfied with the amount of time I spent per module	*- How much time did you spent per module?*<br>*- What was the most time-consuming?*<br>*- Would you have liked modules to be more/less time-consuming?*
8. The face-to-face contact with my personal coach, aside from the online period, was desirable	*- Why was this (not) desirable?*<br>*- What was the added value?*
9. I was satisfied with the goal-setting together with my coach	*- Why where you (not) satisfied*<br>*- What could be improved to make it easier?*
10. The oral explanation of the website was clear to me	*- Points for improvement?*
11. The written explanation of the website was clear to me	*- Points for improvement?*
12. I was satisfied with the amount of time between modules (1-2 weeks)	*- Longer or shorter and why?*
13. I was satisfied with the number of modules (4)	*- More or less and why?*
14. I was satisfied with the structure of the modules (video clip, education, self-refection assignment, 5-stepplan)	*- Did it feel natural to follow the steps in this order?*<br>*- Why (not)?*<br>*- Points for improvement?*
15. I made use of:	
15a. The video-clips	*- Why (not)?*<br>*- Why did it (not) appeal to you?*
15b. The educational introductions	*- Why (not)?*<br>*- Why did it (not) appeal to you?*
15c. The self-reflection assignments	*- Why (not)?*<br>*- Why did it (not) appeal to you?*
15d. The 5-step change plans	*- Why (not)?*

	- Why did it (not) appeal to you?
15e. The discussion forum	*- Why (not)?*<br>*- Reading/posting?*<br>*- Why did it (not) appeal to you?*
15f. The 'help' button	*- Why (not)?*<br>*- Why did it (not) appeal to you?*
	Were there components you would omit or add to the program?
16. I have experienced privacy issues:	
16a. In general	*- What made you feel (un)safe?*<br>*- How could this be improved?*
16b. In communication with my coach	*- What made you feel (un)safe?*<br>*- How could this be improved?*
16c. At the discussion forum	*- What made you feel (un)safe?*<br>*- How could this be improved?*
17. In general, I am satisfied with the program	*- Why (not)?*<br>*- Points for improvement?*
18. The modules I choose were useful for me	*- Why (not)?*<br>*- Points for improvement?*
19. The content of the modules was interesting	*- Why (not)?*<br>*- Points for improvement?*
20. The tone of the text was appealing to me	*- Why (not)?*<br>*- Points for improvement?*
21. I used the information that was offered in the program in my daily life	*- Why (not)?*<br>*- Points for improvement?*
22. After following the program, I am more confident that me and my family member can cope with the memory impairment	*- Why (not)?*<br>*- Points for improvement?*
23. The video clips were a valuable addition to the program	*- Why (not)?*<br>*- Points for improvement?*
24. The written introduction was informative and helpful	*- Why (not)?*<br>*- Points for improvement?*
25. The self-reflection assignments helped me to carefully review my own situation	*- Why (not)?*<br>*- Points for improvement?*
26. The 5-stepplan helped me to systematically tackle situations that I wished to change	*- Why (not)?*<br>*- Points for improvement?*
27. The discussion forum was a valuable addition to the program	*- Why (not)?*<br>*- Points for improvement?*
	Which aspect(s) of the program were most/least helpful to you?

28. After following the program, I know how to tackle unwanted situations in the future more easily	- *Why (not)?*<br>- *Points for improvement?*
29. I would recommend the program to other family members of people with memory impairment	- *Why (not)?*<br>- *What aspects of the program would you recommend in particular?*
30. Remaining remarks?	
